# Just a click away: Action–state orientation moderates the impact of task interruptions on initiative

**DOI:** 10.1111/jopy.12498

**Published:** 2019-07-06

**Authors:** Max V. Birk, Regan L. Mandryk, Nicola Baumann

**Affiliations:** ^1^ Department of Industrial Design Eindhoven University of Technology Eindhoven Netherlands; ^2^ Department of Computer Science University of Saskatchewan Saskatoon Canada; ^3^ Department of Psychology University of Trier Trier Germany

**Keywords:** action versus state orientation, daily hassles, initiative versus hesitation, intention–behavior gap, Personality Systems Interactions (PSI) theory, task interruption

## Abstract

**Objective:**

The present research examines the role of individual differences in self‐regulation (i.e., demand‐related action–state orientation) on initiative to resume an interrupted task.

**Method:**

In three studies (*N*_1_ = 208, 55% male, *M*_age_ = 33.2; *N*_2_ = 457, 62% male, *M*_age_ = 31.7; *N*_3_ = 210, 60% male, *M*_age_ = 32.6), participants were notified about a network interruption while playing a computer game. Participants could dismiss the interrupting notification by clicking a continue button or wait until the notification timed out. We manipulated demand by presenting notifications *during* (demand) versus *after* game rounds (no demand).

**Results:**

Demand‐related action orientation was associated with higher probability to dismiss the notification during a game round, controlling for dismissal after a game round. Findings occurred when controlling for task ability and task motivation, were specific for demand‐ and not threat‐related action orientation, were complemented by shorter dismissal latencies, and were stable across interruption timeouts (Studies 1–3). Exposure through repetition resulted in adaptation (Study 3).

**Conclusion:**

The findings suggest that people with lower action orientation have less self‐regulatory ability to initiate goal‐directed action and resume interrupted tasks—even if they are just a click away. Findings are discussed within the framework of Personality Systems Interactions theory.

## 1 INTRODUCTION

From printer jams to traffic jams, life is full of daily hassles that interrupt our goal‐directed behavior. How we handle small hassles and interruptions has implications on how we function, including effects on our mood (DeLongis, Folkman, & Lazarus, 1988), cognitive performance (Louch, O'Hara, Gardner, & O'Connor, 2017; Sliwinski, Smyth, Hofer, & Stawski, 2006), and ultimately on our well‐being and health (Aldwin, Jeong, Igarashi, Choun, & Spiro, 2014; Asselmann, Wittchen, Lieb, & Beesdo‐Baum, 2017; Charles, Piazza, Mogle, Sliwinski, & Almeida, 2013). This makes interruptions a potent stressor at the workplace and elsewhere. Specifically, interruptions are a type of demand (i.e., a central component of stress) that draws on our ability to initiate goal‐directed behavior to resume the task. Individual differences in demand‐related action orientation (i.e., the ability to self‐regulate affect under demanding conditions) should therefore affect how we manage interruptions (Diefendorff, Richard, & Gosserand, 2006; Kuhl, 2000, 2001; Kuhl & Beckmann, 1994b).

In applied settings, it is often hard to tell why some individuals are better at initiating action to resume an interrupted task than others: Are they more motivated for the primary task (motivation) or better able to regulate themselves (volition)? To better understand the determinants of initiative, we presented an interrupting system notification during computer use that required no action other than to dismiss the notification. We created a subtle manipulation of demand by presenting notifications *during* (demand) versus *after* tasks (no demand). Then, we assessed participants' self‐regulatory abilities to initiate by measuring whether or not they clicked on the “continue” button, which dismissed the notification and allowed the player to resume, and measuring the time between presenting the interruption and clicking the button. We expected action‐ compared to state‐oriented individuals to show more initiative under this small‐scale demand that people experience frequently in daily life. Such a differential effect would emphasize volitional over motivational determinants of coping with interruptions. From an applied point of view, understanding the determinants of coping with interruptions would be informative because training self‐regulatory abilities (volition) requires a different type of intervention compared to the enhancement of task motivation (e.g., Kuhl, Kazén, & Koole, 2006; Kuhl & Quirin, 2011).

### 1.1 Action–state orientation

The personality disposition of action versus state orientation captures individual differences in self‐regulation. Demand‐related action orientation is the ability to self‐generate positive affect, to easily decide between action alternatives, and to quickly initiate the implementation of intentions under demanding conditions (Jostmann & Koole, 2007; Kazén, Kaschel, & Kuhl, 2008; Kuhl, 2000, 2001; Kuhl & Beckmann, 1994b; Ruigendijk, Jostmann, & Koole, 2018). Thus, action orientation bridges the often substantial gap between intention and behavior (Webb & Sheeran, 2006). In contrast, demand‐related state orientation (i.e., low action orientation) is the low ability to self‐generate positive affect and is associated with indecisiveness and hesitation under demanding conditions. Threat‐ or failure‐related action versus state orientation is the high versus low ability to self‐regulate negative affect, to disengage from failure, and to maintain self‐access in the face of threats (Baumann, Kaschel, & Kuhl, 2007; Kuhl & Beckmann, 1994a). Although both dimensions are often highly correlated, demand‐ rather than threat‐related action–state orientation moderates the impact of demanding conditions on initiative. Whereas demanding conditions impair state‐oriented individuals' initiative, they do not impair—and can sometimes even improve—action‐oriented individuals' initiative (Koole, Jostmann, & Baumann, 2012).

Maintaining initiative in the face of demands is important for the attainment of personal goals. In line with this, action orientation has been found to be associated with lower procrastination as assessed by self‐report and behavioral indicators, such as meeting deadlines (Beswick & Mann, 1994; Blunt & Pychyl, 1998), increases in positive affect and energy over the course of a semester (Brunstein, 2001), higher rates of self‐reported goal attainment in daily life (Diefendorff et al., 1998), better adherence to exercise intentions (Kendzierski, 1990), and higher control of eating behavior (Palfai, 2002). Thus, the benefits of action orientation are evident across many important life domains (Koole et al., 2012; Kuhl & Beckmann, 1994b).

Action orientation is particularly important when goal‐directed action is not triggered by external cues but has to be self‐initiated (Graf & Uttl, 2001). For example, Dibbelt (1997) and Kazén et al. (2008, Exp. 2) found that action‐ and state‐oriented individuals had similar response latencies for initiating simple motor actions (i.e., moving a cursor to one of two possible targets) when there was a clear external cue (i.e., when one target was closer). In contrast, when there was no external cue (i.e., when both targets were equally far away) and participants had to choose the target by themselves, state‐oriented participants had significantly increased response latencies compared to action‐oriented participants. Moreover, state‐oriented participants' deficits in self‐initiating goal‐directed action emerged only under demanding conditions (Dibbelt, 1997; Kazén et al., 2008).

State‐oriented participants do not only hesitate when two options are equally attractive but also when a clearly preferred action alternative is just a click away (Kuhl & Beckmann, 1994a). During a waiting period, participants could choose between watching recordings of lottery drawings of previous years and an interesting travel documentary. Although post hoc ratings indicated that all participants clearly preferred the travel documentary, many state‐oriented participants did not switch channels, demonstrating lower initiative. Moreover, this lower initiative was only observed under demanding conditions. In contrast, almost all action‐oriented participants initiated the switch to the preferred travel documentary regardless of previous task conditions. Taken together, these findings support the assumption that action–state orientation is about the self‐regulatory ability to initiate action under demand (rather than task motivation per se).

### 1.2 Task interruption creates a demand

The reviewed findings show that differences in self‐regulatory ability between action‐ and state‐oriented participants are evident only under demanding conditions. The term “demanding conditions” refers to a broad range of conditions under which goal‐directed behavior becomes difficult. Examples of demanding conditions are difficult life circumstances (cf. Koole et al., 2012), high cognitive load (Kaschel, Kazén, & Kuhl, 2016), tempting distractors (Baumann & Kuhl, 2005), listlessness (Kazén et al., 2008, Exp. 1), and uncompleted intentions (Goschke & Kuhl, 1993; Kazén et al., 2008, Exp. 2). Even something as simple as a task interruption creates a demand and has been used as an experimental method to induce uncompleted intentions since the early days of Kurt Lewin and his associates (Lewin, 1935, 1936; Ovsiankina, 1928; Zeigarnik, 1927).

Recent studies in applied settings further corroborate the assumption that interruptions are more demanding when occurring during tasks rather than after or between tasks. Monk, Boehm‐Davis, and Trafton (2002), for example, found participants to be more disrupted (as indicated by a slower task resumption) when a secondary task interrupts the middle or the end of a primary task than when presented just before the beginning of a new task. Bailey and Konstan (2006) extend these findings by showing that interruptions during tasks not only increase completion times for the primary task, but also elicit more annoyance and anxiety than interruptions in between tasks. Kazén et al. (2008, Exp. 2) interrupted an ongoing task and manipulated demands simply by telling participants either that they were done with the task (no demand) or that they would continue the task later on (demand). In the demand condition, state‐ but not action‐oriented participants exhibited self‐initiation deficits in the subsequent task. There was no investigation of how resumption of the primary task was differentially affected. To our knowledge, no study investigated the role of action–state orientation on task resumption.

In light of the small scale of demands (e.g., an interruption during a task) that suffice to disrupt volitional action control in state‐oriented individuals, it is important to look for factors that can reduce the impact of such demands and improve initiative. Training self‐regulatory competence would be the method of choice to improve initiative in the long run and across contexts (Kaschel & Kuhl, 2004; Kuhl et al., 2006; Martens & Kuhl, 2004). In addition, task repetition is a simple feature that may offer some short‐term improvements in a given context. For example, findings by Monk (2004) show that participants resume a primary task faster when interrupted more frequently (every 10 vs. 30 s). Furthermore, findings by Trafton, Altmann, Brock, and Mintz (2003) indicate that adaptation to particularly disruptive forms of interruption may occur very fast: when interrupted a second time during a task without warning, participants already resumed the primary task as fast as participants who received a preparatory warning signal. Therefore, it is informative to test whether repetition also helps state‐oriented individuals to adapt and how fast adaptation occurs.

### 1.3 The present research

In the present research, we conducted three studies to examine whether demand‐related action–state orientation moderates the effects of task interruption on initiative, operationalized as the active dismissal of a notification in order to resume an interrupted task. Previous work has focused on more pronounced manipulations of demand, and our goal was to create a demand that was decidedly trivial, but through frequent and persistent occurrence over time, might perhaps not be trivial at all. We had participants engage in a motivating task (playing a custom‐built clone of a popular computer game), and we presented a notification in the form of a simulated network interruption that paused game play. Participants could dismiss the notification by clicking a button or could wait until the notification timed out to continue playing. As a primary task, we chose a game that is known to be engaging to increase the likelihood that participants would be intrinsically motivated to resume the task and would desire to dismiss the notification. Games offer significant motivational pull and are able to satisfy basic psychological needs to experience competence, autonomy, and relatedness (Ryan, Rigby, & Przybylski, 2006).

We experimentally manipulated demand by presenting the notification *during* a game round (demand) versus *after* a game round (no demand). Note that although the latter does not interrupt an ongoing game round, it still interrupts the game—albeit at a more natural point for a break. We assessed initiative by measuring clicking (yes vs. no) and latencies (in seconds) for selecting the “continue” button, which dismissed the notification and allowed the player to resume. Our hypothesis was that action orientation would predict higher initiative when interrupted during a game round, when controlling for initiative after a game round.

Note that there are multiple factors that may influence whether individuals click on the continue button or not (e.g., familiarity with computers, impulsiveness, lack of patience, need for a break, interest in the game). However, all of these factors should have the same effect for interruptions during and after tasks. In contrast, our hypothesis for action orientation is specific for interruptions during a task, when controlling for interruptions after a task. Furthermore, interruptions during a task create a demand rather than a threat. Therefore, we expected demand‐ but not by threat‐related action orientation to predict initiative.

## 2 STUDY 1

In Study 1, our goal was to demonstrate the differential effect of action–state orientation when interrupting participants during a game round. We developed a clone of the popular Match‐3 game Bejeweled (PopCap, http://www.bejeweled.com) that requires constant attention and is known to be engaging, which helps to ensure that the interruption is meaningful. We interrupted participants twice with a simulated network interruption notification: during and after a game round. Participants could either dismiss the notification or wait 60 s for the notification to time out and return to the game. We expected demand‐related action orientation to increase the probability of dismissal during a game round when controlling for dismissal after a game round.

In the analyses of dismissal latencies, we had two strategies for handling nondismissal during the 60 s period. Our first strategy was to set latencies for nondismissal to 60 s in order to have a more continuous (rather than binary) measure of initiative across the full sample. We expected action orientation to be associated with higher initiative (i.e., shorter latencies during a game round when controlling for latencies after a game round). Our second strategy was to exclude all participants who did not dismiss both notifications (during and after a task) to further explore the decision process. We expected that action orientation is not associated with a more deliberate and time‐consuming decision process.

### 2.1 Methods

#### 2.1.1 Participants

A power analysis was conducted prior to data collection using G*Power 3 (Faul, Erdfelder, Lang, & Buchner, 2007). In a related study (Kazén et al., 2008, Exp. 2, ∆*R* ^2^ = 0.06), the action orientation and demand interaction accounted for a small (0.02) to medium (0.13) amount of variance (see Cohen, 1988, pp. 414–416). We assumed a conservative, small effect size (*f* = 0.10) for the expected interaction and used a power of 0.80. The power analysis suggested a total sample size of 200 participants. We invited a slightly higher number of participants (*N* = 230) to compensate for potential drop‐out. From this sample, 22 participants were excluded from further analysis as recommended in best practices for online behavioral studies (e.g., Mason & Suri, 2012) for incomplete data, for indicating that the instructions were confusing, and for low compliance, defined as responding incorrectly to test questions (e.g., “interruptions were caused by network problems?”), showing results that differed from the mean by more than three standard deviations, and/or having a completion time for questionnaires that was one standard deviation below mean completion time (which was not possible if reading the questions and considering the answers). Two hundred and eight participants (115 male, 87 female, 6 preferred not to answer) with a mean age of 33.23 years (*SD* = 10.04, min = 19, max = 69 years) remained. Participants played games frequently (94.2% played a few times a month or more frequently).

#### 2.1.2 Experimental task

We developed a clone of Bejeweled (PopCap, http://www.bejeweled.com/), a game in which the goal is to swap adjacent gems to align gems of the same color. The game is easy to learn and there is a high degree of familiarity with the game mechanics in the general population, as many popular casual games are variants of the match‐3 game mechanic (e.g., Candy Crush Saga, King, http://candycrushsaga.com). We presented participants with an 8 by 8 grid of gems in one of five colors. Participants clicked or dragged adjacent gems to swap their locations and make matches. Participants gained 5 points for each gem when 3 or more gems of the same color were aligned. While three‐of‐a‐kind matches only gave points, four‐of‐a‐kind matches created special pieces that destroyed all adjacent pieces; five‐of‐a‐kind matches in “L,” “T,” or “+”‐shapes created special pieces that destroyed gems in a horizontal and vertical line; five‐of‐a‐kind matches in one line destroyed all gems in the matched color. We instructed participants on how to play the game using an avatar, who was present during game play and the simulated network interruption. We allowed half of the participants to customize their avatar as to foster motivation (Birk, Atkins, Bowey, & Mandryk, 2016); however, this manipulation did not affect any reported dependent measure or interact with any measures in our analyses, and so we do not consider it further in this paper. To further the illusion that they were playing over a network, we started each game round with a loading icon notifying them that they were “connecting to other players”; this message was displayed for a random duration between 3 and 5 s. We additionally presented a simulated leaderboard after each round showing their relative performance to other players, using the method described in Bowey, Birk, and Mandryk (2015); participants were always randomly placed in a neutral position, that is, position 10–15 out of 32 positions.

#### 2.1.3 Procedure

**Figure 1:**
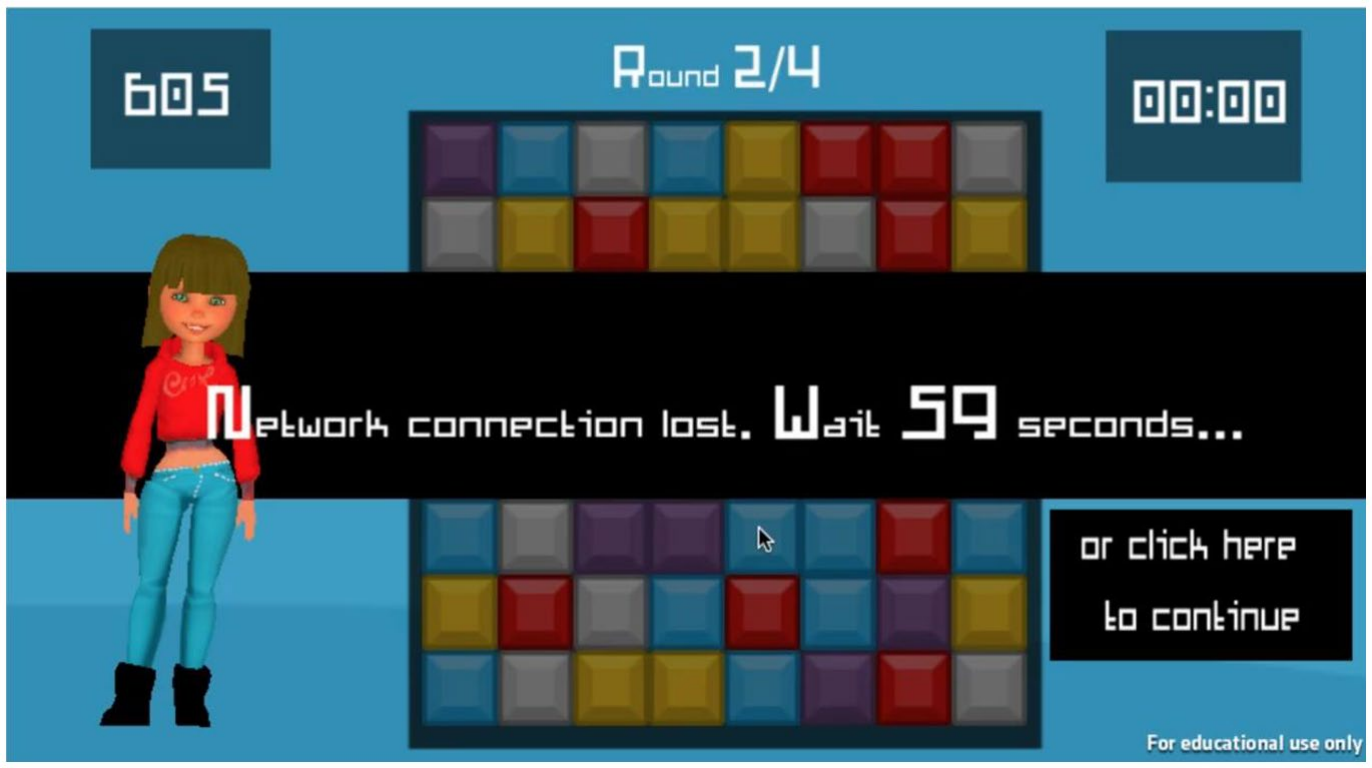
Game interface with network connection message (timer counts down to zero) and the “click here to continue” button to dismiss the interrupting notification [Color figure can be viewed at https://www.wileyonlinelibrary.com]

Participants were recruited through Amazon's Mechanical Turk (MTurk), which matches workers to requesters of work through an online platform. MTurk has been shown to be robust for conducting experiments with human participants (Crump, McDonnell, & Gureckis, 2013; Kittur, Chi, & Suh, 2008) when precautions are put into place, such as including attention‐testing questions and filtering outlier participants or those who show very little variance on multiple items of a validated scale (Mason & Suri, 2012; Meade & Craig, 2012). After giving consent, participants provided answers to a series of validated scales measuring trait attributes, and demographic questions on their game‐playing experience and preferences. After completing the demographic questionnaires, participants were instructed on how to play the game and then played four 1‐min rounds of Bejeweled. Participants were interrupted twice—once during play (demand) and once after play (no demand)—in Round 2 and Round 4. The interruption was the notification “Network connection lost. Wait 60 s…” in which the 60 counted down to zero. Participants could also dismiss the interruption by clicking on the “or click here to continue” button located below the interrupting notification (see Figure 1). To counterbalance the order of presentation of demand, half of the participants received the demanding condition in Round 2, whereas the other half received it in Round 4. Following completion of the experimental block, participants completed several validated scales about their experience. Finally, we gave participants the opportunity to provide a free‐form text response about their perceived purpose of the experiment and we debriefed participants about the experiment and ensured they understood the purpose through a series of manipulation check questions with binary responses. Ethical approval was obtained from the behavioral research ethics board of the University of Saskatchewan, and participants were asked to give informed consent.

#### 2.1.4 Action orientation

The Action Control Scale (ACS; Kuhl, 1994) was used to assess demand‐related (AOD; 12 items, Cronbach's *α* = 0.85) and threat‐related (AOT; 12 items, Cronbach's *α* = 0.88) action orientation. An example item for AOD is “When I am facing a big project that has to be done: (a) I often spend too long thinking about where I should begin, or (b) I don't have any problems getting started.” An example item for AOT is “When I have lost something that is very valuable to me and I can't find it anywhere: (a) I have a hard time concentrating on something else, or (b) I put it out of my mind after a little while.” In both items, options “a” represent the state‐oriented and options “b” the action–oriented response alternatives. In each scales, action‐oriented response alternatives were counted so that the scale ranged from 0 to 12, with lower scores indicating state orientation (i.e., action orientation) and higher scores indicating action orientation. For further information on reliability and validity of the scale, see Kuhl and Beckmann (1994b) and Diefendorff, Hall, Lord, and Strean (2000).

#### 2.1.5 Motivation

We measured motivation to play using the 18‐item Intrinsic Motivation Inventory (McAuley, Duncan, & Tammen, 1989), which measures intrinsic motivation related to a task through the four dimensions: interest–enjoyment (5 items, Cronbach's *α* = 0.86), perceived competence (5 items, Cronbach's *α* = 0.89), effort–importance (4 items, Cronbach's *α* = 0.76), and pressure–tension (4 items, Cronbach's *α* = 0.86). Agreement with items was assessed using a 5‐point scale (1 = strongly disagree, 2 = disagree, 3 = neutral, 4 = agree, 5 = strongly agree). The IMI has previously been used to describe game experience (cf. Birk et al., 2016; Ryan et al., 2006). Consistent with previous work, we changed “task” to “game” in the phrasing of the questions—e.g., “I enjoyed this game very much,” “I think I am pretty good at this game,” “I felt pressured while playing this game,” and “I tried very hard while playing the game”—to ensure that participant ratings were related to the game and not the experimental context. We measured motivation to evaluate the enjoyment of the game itself and to rule out motivational factors as an explanation for differential effects in click behaviors based on action–state orientation.

#### 2.1.6 Demographics

We collected the participant's age as a continuous variable, self‐reported gender (female, male, transgender, prefer not to answer), and gaming experience (7 = every day, 5 = a few times per week, 3 = a few times per month, 1 = a few times per year, 0 = not at all).

### 2.2 Results

#### 2.2.1 Descriptive information

Correlations, means, and standard deviations are listed in Table 1. Demand‐related action orientation correlated positively with threat‐related action orientation and with interest and enjoyment in the game. Overall, 91.59% of the participants dismissed notifications. Dismissal—aggregated across notifications during and after a task—did not correlate with any of the study variables.

**Table 1:** Correlations, means, and standard deviations in Study 1 (upper right; *N*_1_ = 208) and Study 2 (lower left; *N*_2_ = 457)

	(1)^a^	(2)	(3)	(4)	(5)	(6)	(7)	(8)	(9)	(10)	(11)^a^	*M*	*SD*
(1) Gender (1 = female, 2 = male)^a^		−0.22**	−0.03	−0.10	−0.19**	−0.01	−0.16*	−0.02	−0.01	−0.16*	−0.05		
(2) Age	−0.12*		−0.15*	−0.12	−0.21**	−0.28**	−0.20**	−0.27**	−0.14*	−0.04	−0.01	33.23	10.04
(3) AOD	−0.01	−0.26**		−0.64**	−0.02	−0.11	−0.20**	0.06	−0.13	−0.11	−0.01	6.99	3.97
(4) AOT	−0.13**	−0.16**	−0.62**		−0.04	−0.16*	−0.05	−0.01	−0.08	−0.06	−0.03	5.75	3.66
(5) Game experience	−0.16**	−0.15**	−0.05	−0.02		−0.23**	−0.01	−0.22**	−0.08	−0.25**	−0.08	6.15	0.95
(6) Performance	−0.19**	−0.24	−0.12*	−0.11*	−0.28**		−0.01	−0.51**	−0.13	−0.23**	−0.11	828	329
(7) Interest and enjoyment	−0.14**	−0.13	−0.18**	−0.09*	−0.01	−0.10*		−0.39**	−0.47**	−0.15*	−0.12	3.84	0.73
(8) Perceived competence	−0.04	−0.07	−0.15**	−0.15**	−0.11*	−0.16**	−0.47**		−0.12	−0.41**	−0.05	3.68	0.81
(9) Effort and importance	−0.01	−0.18**	−0.18**	−0.00	−0.07	−0.12*	−0.38**	−0.08		−0.11	−0.01	3.88	0.70
(10) Tension and pressure	−0.08	−0.01	−0.24**	−0.31**	−0.04	−0.06	−0.13**	−0.42**	−0.27**		−0.02	2.40	0.99
(11) Dismissal (%)^a^	−0.03	−0.15**	−0.01	−0.01	−0.04	−0.24**	−0.04	−0.04	−0.04	−0.03		91.59	24.35
Scale			0–12	0–12	0–7		1–5	1–5	1–5	1–5			
*M*		31.73	6.93	5.61	6.03	870	3.79	3.28	4.14	2.99	70.02		
*SD*		9.93	3.87	3.65	0.96	342	0.74	0.84	0.66	0.70	39.99		

Note

AOD, demand‐related action orientation; AOT, threat‐related action orientation.

a Spearman's *ρ*.

* *p* < .05;

** *p* < .01.

#### 2.2.2 Manipulation check

An exact McNemar's test determined that there was a significant difference in the proportion of participants who dismissed notifications during a task (89.42%) compared to after task (93.75%), *p* = .035. The finding is consistent with the assumption that task interruption does indeed create a demand that interferes with active task resumption.

#### 2.2.3 Dismissal

To test whether demand‐related action orientation predicted initiative under demanding conditions, we conducted a binary logistic regression analysis with dismissal (0 = no, 1 = yes) during a task as the dependent variable. In Step 1, we controlled for dismissal after a task (as a baseline) and order of the two interruptions (after/during vs. during/after). In Step 2, we entered gaming experience, game performance score, and four motivational variables. In Step 3, we entered demand‐related action orientation. Results are listed in Table 2. Dismissal after a task was significantly associated with dismissal during a task, *B* = −3.89, *SE* = 0.74, Wald (1) = 27.74, *p* < .001, OR = 0.02. Step 1 significantly improved the goodness of fit compared to a model containing only the constant, *χ^2^* (2) = 39.20, *p <* .001. Gaming experience, performance, and motivational variables were not significantly associated with dismissal during a task. Step 2 did not significantly improve the goodness of fit, *χ^2^* (6) = 4.27, *p* = .640. Consistent with expectations, demand‐related action orientation was significantly associated with dismissal during a task, *B* = 0.17, *SE* = 0.08, Wald (1) = 4.62, *p* = .042, OR = 1.19. The odd ratio (OR) of 1.19 indicates that a one unit increase in demand‐related action orientation corresponded to a 1.19 times (19%) higher probability of dismissing the notification during a task. Step 3 significantly improved the goodness of fit, *χ^2^* (1) = 4.96, *p* = .026. Overall, the model explained 42% of variance in dismissal versus nondismissal.

**Table 2:** Binary logistic regression analyses predicting dismissal versus nondismissal of the notification during a task

	Study 1					Study 2					Study 3, Task #3				
	*R* ^2^	*χ^2^*	*B*	*SE*	OR	*R* ^2^	*χ^2^*	*B*	*SE*	OR	*R* ^2^	*χ^2^*	*B*	*SE*	OR
Step 1	0.35	39.20***				0.36	134.17***				0.32	54.85***			
Dismissal after task			−3.89***	0.74	0.02			−2.76***	0.27	0.06			−2.33***	0.34	0.10
Order			−0.98	0.59	0.38			−0.90***	0.26	0.41					
Step 2	0.38	4.27				0.38	12.31				0.33	2.32			
Gaming experience			−0.13	0.33	0.88			−0.14	0.13	1.15			−0.02	0.17	0.98
Performance			−0.00	0.00	1.00			−0.00	0.00	1.00			−0.00	0.00	1.00
Interest/enjoyment			−0.91	0.57	0.40			−0.13	0.21	0.88			−0.13	0.22	1.14
Perceived competence			−0.04	0.45	0.96			−0.07	0.18	1.07			−0.04	0.25	0.96
Effort/importance			−0.51	0.47	1.66			−0.16	0.22	1.17			−0.01	0.25	0.99
Tension/pressure			−0.40	0.34	0.67			−0.05	0.15	1.05			−0.15	0.20	0.87
Step 3	0.42	4.96*				0.40	5.02*				0.35	4.58*			
Action orientation (AOD)			−0.17*	0.08	1.19			−0.08*	0.03	1.08			−0.10*	0.05	1.11
Classification accuracy	93.80%					80.30%					77.50%				
*N*	208					457					210				

Note

*R* ^2 ^= Nagelkerke's *R^2^*; OR, odd ratio; AOD, demand‐related action orientation.

* *p* < .05;

*** *p* < .001.

**Figure 2:**
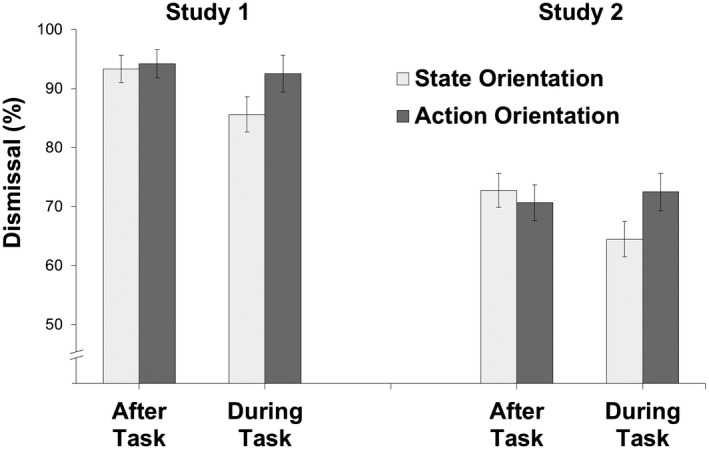
Initiative (dismissal of notification) as a function of demand (interruption during vs. after task) and demand‐related action–state orientation in Study 1 (*N* = 208; notification timeout: 60 s) and Study 2 (*N* = 457; notification timeout: 10 s)

In an additional binary logistic regression analysis, we entered threat‐ instead of demand‐related action orientation in Step 3 as a test of discriminant validity. Consistent with expectations, threat‐related action orientation was not associated with a higher probability of dismissing the notification during a task, *B* = 0.14, *SE* = 0.08, Wald (1) = 2.72, *p* = .099, OR = 1.15. Step 3 did not significantly improve the goodness of fit, *χ^2^* (1) = 2.93, *p* = .087. Thus, the effect is specific for demand‐related action orientation and occurred over and above of gaming experience, performance, and motivational variables. To further illustrate the finding (see left side of Figure 2), we graphed dismissal rates during and after tasks using a median split to classify participants as state‐oriented (*n* = 107, scores 0–7) and action‐oriented (*n* = 101, scores 8–12).

#### 2.2.4 Latency

To create a more continuous measure of initiative, we set nondismissals to 60 s and conducted a hierarchical regression analysis on latencies (*M* = 10.38 s, *SD* = 13.96). As listed in Table 3, the analysis yielded highly significant effects of latencies after a task, *β* = 0.51, *t*(1, 205) = 8.68, *p* < .001, and order, *β* = −0.25, *t*(1, 205) = −4.19, *p* < .001. Gaming experience, performance, and motivational variables were not significantly associated with dismissal latencies during a task. Consistent with expectations, demand‐related action orientation was associated with faster dismissal during a task, *β* = −0.15, *t*(1, 198) = −2.46, *p* = .015, and accounted for additional variance, ∆*R* ^2^ = 0.020, ∆*F*(1, 198) = 6.06, *p* = .015. Consistent with expectations, there was no significant effect when entering threat‐ instead of demand‐related action orientation, *β* = 0.04, *t*(1, 198) = 0.57, *p* = .569. Findings indicate that this continuous measure of initiative (dismissal latencies) yielded effects that were similar to the binary measure (dismissal).

**Table 3:** Hierarchical regression analyses predicting latency for dismissal during a task with two strategies for handling nondismissal (ND)

	Study 1				Study 2				Study 3, Task #3			
	ND = 60 s		ND excluded		ND = 10 s		ND excluded		ND = 10 s		ND excluded	
	∆*R* ^2^	*β*	∆*R* ^2^	*β*	∆*R* ^2^	*β*	∆*R* ^2^	*β*	∆*R* ^2^	*β*	∆*R* ^2^	*β*
Step 1	0.30***		0.03		0.35***		0.32***		0.23***		0.06*	
Dismissal latency after		−0.51***		−0.04		−0.59***		−0.25***		−0.50***		0.25*
Order		−0.25***		−0.16		−0.39***		−0.66***				
Step 2	0.01		0.04		0.02*		0.01		0.00		0.04	
Gaming experience		−0.02		−0.09		−0.05		−0.06		−0.01		−0.07
Performance		−0.04		−0.10		−0.13*		−0.04		−0.05		0.04
Interest/enjoyment		−0.11		−0.02		−0.07		−0.10		−0.02		−0.13
Perceived competence		−0.03		−0.14		−0.05		−0.08		−0.02		−0.11
Effort/importance		−0.03		−0.01		−0.06		−0.06		−0.01		0.04
Tension/pressure		−0.10		−0.09		−0.01		−0.04		−0.03		−0.12
Step 3	0.02*		0.00		0.01*		0.01		0.02*		0.04	
Action orientation (AOD)		−0.15*		−0.03		−0.11*		−0.09		−0.13*		−0.22
Total *R^2^*	0.33***		0.07*		0.38***		0.34***		0.25***		0.14*	
*N*	208		183		457		274		210		88	

Note

AOD, demand‐related action orientation.

* *p* < .05;

*** *p* < .001.

To further explore the decision process, we excluded the 25 participants who did not dismiss both notifications. In the subsample of *N* = 183 who dismissed notifications, we conducted the same hierarchical regression analyses on latencies (*M* = 5.59 s, *SD* = 6.32). As listed in Table 3, the analysis did not yield any significant effects. Consistent with expectations, demand‐related action orientation was not associated with significantly *slower* dismissal during a task, *β* = 0.03, *t*(1, 173) = 0.40, *p* = .688, indicating that decision processes were not more deliberate.

### 2.3 Discussion

The results of our first experiment show that action orientation was associated with a higher probability of dismissing the notification during a task. The finding is consistent with the assumption that action‐oriented participants are better able to initiate goal‐directed action under demand. We controlled for several alternative explanations. First, we controlled for dismissal after a task (i.e., under no demand). Although many factors may influence clicking the continue button (e.g., impulsiveness, lack of patience, familiarity with computers and online ads), none of these can easily explain the difference in probability of dismissing an interruption during versus after a task.

Second, the effect of action orientation occurred when controlling for the effects of *task ability* (i.e., gaming experience, performance) and *task motivation* (i.e., interest/enjoyment, perceived competence, effort/importance, tension/pressure) in the regression model. Third, the effect occurred for demand‐related (self‐regulation of positive affect) but not threat‐related action orientation (self‐regulation of negative affect). When a subtle demand hampers positive affect, demand‐related action orientation helps to self‐generate the positive affect needed to initiate the necessary action for task resumption. Fourth, the effect held across binary (dismissal vs. nondismissal) and continuous (dismissal latency) measures of initiative. Finally, action‐oriented participants did not engage in more deliberate and time‐consuming decision processes. These results support our hypothesis that action orientation is associated with greater self‐regulatory ability to initiate action under demand, even when the demand itself is subtle and the measurement of initiative is fine grained.

Because the notification was presented for 60 s, we observed a very high probability of dismissal (92%). This ceiling effect indicates that we created a situation in which most participants eventually clicked the continue button. In Study 2, we therefore aimed at creating a situation that yields probabilities for dismissal closer to 50% by setting the notification timeout around the mean (*M* + 1 *SD*) of dismissal latencies in Study 1 (i.e., 10 s).

## 3 STUDY 2

The goal of Study 2 was to replicate and extend our results from Study 1. First, we further improved our nuanced measurement of initiative. By shortening the notification timeout from 60 to 10 s, we aimed at reducing the ceiling effect. Note that the demand is the same as in Study 1 (i.e., an interruption during an ongoing task round). Second, we obtained a highly significant order effect in dismissal latencies in Study 1. Therefore, we roughly doubled the number of participants to be able to detect the expected effect with a small effect size over and above order effects.

### 3.1 Methods

The methods for Study 2 were identical to those of Study 1, except that the interrupting notification was set to count down from 10 (rather than 60) s before automatically being dismissed. Internal consistencies (Cronbach's *α*) were sufficient for demand‐ and threat‐related action orientation (0.85, 0.89) and the four motivational variables of interest/enjoyment (0.86), perceived competence (0.88), effort/importance (0.82), and tensions/pressure (0.86).

#### 3.1.1 Participants

Five hundred participants were recruited through Amazon Mechanical Turk. From this sample, 43 were removed from subsequent analyses as described in Study 1, leaving 457 participants (203 female; mean age = 31.73, *SD* = 9.93, min = 18, max = 67 years). Participants were again familiar with playing digital games (91.9% played a few times a month or more frequently). Data were treated in the same way as in Study 1.

### 3.2 Results

#### 3.2.1 Descriptive information

As listed in Table 1, demand‐related action orientation correlated positively with threat‐related action orientation, interest/enjoyment, perceived competence, effort/importance, and negatively with performance as well as tension/pressure. Overall, 70.02% of the participants dismissed notifications. Dismissal correlated negatively with age and positively with performance.

#### 3.2.2 Manipulation check

An exact McNemar's test determined that there was only a marginally significant difference in the proportion of participants who dismissed notifications during a task (68.27%) compared to after a task (71.77%), *p* = .059. The finding is not consistent with the assumption that task interruption creates a demand that interferes for all participants with active task resumption.

#### 3.2.3 Dismissal

We conducted a binary logistic regression analysis with dismissal versus nondismissal of the notification during a task as the dependent variable (see Table 2). Dismissal after a task was significantly associated with dismissal during a task, *B* = −2.76, *SE* = 0.27, Wald (1) = 104.56, *p* < .001, OR = 0.06. Participants were more likely to dismiss notifications during a task when notified for the second time (order: after/during) compared to the first time (order: during/after), *B* = −0.90, *SE* = 0.26, Wald (1) = 11.91, *p* < .001, OR = 0.41. Step 1 significantly improved the goodness of fit compared to a model containing only the constant, *χ^2^* (2) = 134.17, *p* < .001. Performance, gaming experience, and motivational variables were not significantly associated with dismissal during a task. Step 2 did not significantly improve the goodness of fit, *χ^2^* (6) = 12.31, *p* = .055. Consistent with expectations, demand‐related action orientation was associated with dismissal during a task, *B* = 0.08, *SE* = 0.03, Wald (1) = 4.94, *p* = .026, OR = 1.08. One unit increase in demand‐related action orientation corresponded to a 1.08 times (8%) higher probability of dismissal during a task. Step 3 significantly improved the goodness of fit, *χ^2^* (1) = 5.02, *p* = .025. Overall, the model explained 40% of variance in dismissal versus nondismissal.

In an additional binary logistic regression analysis, we entered threat‐ instead of demand‐related action orientation in Step 3. Consistent with expectations, threat‐related action orientation was not associated with dismissal of notifications during a task, *B* = 0.01, *SE* = 0.04, Wald (1) = 0.06, *p* = .802, OR = 1.01, and did not significantly improve the goodness of fit, *χ^2^* (1) = 0.06, *p* = .802. Thus, the effect is specific for demand‐related action orientation and occurred when controlling for gaming experience, performance, and motivational variables. To further illustrate the finding (see right side of Figure 2), we graphed dismissal rates during and after tasks using a median split to classify participants as state‐oriented (*n* = 240, scores 0–7) and action‐oriented (*n* = 217, scores 8–12).

#### 3.2.4 Latency

When setting latencies for nondismissal to 10 s, average dismissal latencies were *M* = 5.28 s (*SD* = 2.93). As listed in Table 3, the hierarchical regression analysis yielded significant effects of dismissal latencies after a task, *β* = 0.59, *t*(1, 454) = 14.70, *p* < .001, order, *β* = −0.39, *t*(1, 454) = −9.65, *p* < .001, and performance, *β* = −0.13, *t*(1, 448) = −3.27, *p* < .001. Gaming experience and motivational variables were not significantly associated with dismissal latencies during a task. Consistent with expectations, demand‐related action orientation was associated with faster dismissal during a task, *β* = −0.11, *t*(1, 447) = −2.20, *p* = .028, and accounted for additional variance, ∆*R* ^2^ = 0.008, ∆*F*(1, 447) = 2.70, *p* = .028. Consistent with expectations, there was no significant effect when entering threat‐ instead of demand‐related action orientation, *β* = 0.09, *t*(1, 447) = 1.89, *p* = .059. Findings indicate that the continuous measure of initiative (dismissal latencies) yielded similar effects to the binary measure (dismissal).

When including only the 247 participants who dismissed both notifications, average dismissal latencies were *M* = 3.17 s (*SD* = 1.16). As listed in Table 3, in this subsample, the same hierarchical regression analysis yielded significant baseline, *β* = 0.25, *t*(1, 271) = 4.07, *p* < .001, and order effects, *β* = −0.66, *t*(1, 271) = −10.96, *p* < .001. No other effects were significant. Consistent with expectations, demand‐related action orientation was not associated with significantly *slower* dismissal during a task, *β* = 0.09, *t*(1, 264) = 1.59, *p* = .113. Findings do not suggest that action‐oriented participants engaged in more deliberative and time‐consuming decision processes.

### 3.3 Discussion

Shortening the time frame for initiative from 60 to 10 s decreased the overall probability of dismissal from 92% in Study 1 to 70% in Study 2. When eliminating the ceiling effect, we replicated the results of our first experiment that action‐oriented participants were more likely to dismiss notifications during a task. Again, we controlled for dismissal after a task (i.e., under low demand) as well as task ability and task motivation. Furthermore, we replicated that the effect is specific for demand‐related rather than threat‐related action orientation. Finally, the findings with dismissal latencies (a continuous measure of initiative) complemented our findings for dismissal and did not suggest that action‐oriented participants engaged in more deliberate decision processes.

In Studies 1 and 2, we demonstrated the detrimental effect of demand‐related state orientation on initiative under a minor demand in the context of computer use. Because of these observed effects, we were next interested in exploring how we can reduce the effect of state orientation on initiative under minor demands. Previous work on task interruption has shown that repetition can foster adaptation (Monk, 2004; Trafton et al., 2003). In a similar vein, work on action–state orientation has shown that frequent exposure to a demand can eliminate differential effects (Jostmann & Koole, 2007). Thus, in our final study we wanted to bring together these two lines of research and test how fast participants adapt when exposed to the interruption more frequently.

## 4 STUDY 3

The goal of Study 3 was to establish how fast people are able to adapt and become initiative under demand. Research has shown that people adapt to interruptions when the frequency of exposure is increased (Monk, 2004; Trafton et al., 2003), and that increased exposure to a demand can facilitate volitional control (Jostmann & Koole, 2007). In the context of our experimental task, we used repeated exposure to the interrupting notification under demand. Our hypothesis was that state‐oriented participants would adapt and display more initiative after repeated exposure to the demand and dismiss notifications during a task as often as action‐oriented participants.

Because our study is the first to test this hypothesis, we did not know how fast adaption would occur. To be on the safe side, we explored several repetitions. In contrast to Studies 1 and 2, all participants experienced an interruption under no demand first (i.e., notification after a game round), followed by five interruptions under demand (i.e., during game rounds), which were presented every second round. We expected that state‐oriented participants are less likely to dismiss than action‐oriented participants under demand. However, we also expected that the differential effect would disappear after repeated exposure.

### 4.1 Methods

The methods were similar to those of Studies 1 and 2, with the exception of the sequence of interruptions. In Study 3, we presented 12 one‐minute rounds of Bejeweled. We interrupted all participants after Round 1 (i.e., no demand) and during Rounds 3, 5, 7, 9, and 11 (i.e., under demand). As in Study 2, the notification counted down from 10 s before being automatically dismissed. Internal consistencies (Cronbach's *α*) were sufficient for demand‐ and threat‐related action orientation (0.85, 0.80) and the four motivational variables of interest/enjoyment (0.92), perceived competence (0.91), effort/importance (0.78), and tensions/pressure (0.83).

#### 4.1.1 Participants

We aimed at a similar sample size as in Study 1 because we held the order of interruptions (after/during tasks) constant across participants. Two hundred and sixteen participants were recruited. From this sample, 6 participants were excluded using the method described in Study 1. The remaining 210 participants (126 male, 84 female) had a mean age of 32.66 years (*SD* = 9.57, range 18–63 years). Participants were again generally familiar with games (90% played games a few times a month or more frequently). Data were treated in the same way as in Studies 1 and 2.

### 4.2 Results

#### 4.2.1 Descriptive information

Demand‐related action orientation correlated positively with threat‐related action orientation (*r* = .57, *p* < .001) and effort/importance (*r* = .28, *p* < .001). Overall, 70.57% (*SD* = 41.32) of the participants dismissed notifications. The overall probability for dismissal versus nondismissal did not correlate with any study variable.

#### 4.2.2 Manipulation check

Because we did not counterbalance the order of presentation, a manipulation check comparing dismissal of notifications during versus after a task would not be appropriate.

#### 4.2.3 Dismissal

We conducted a binary logistic regression analyses with dismissal versus nondismissal when interrupted during Task #3 as the dependent variable (see Table 2). Dismissal after Task #1 was significantly associated with dismissal during Task #3, *B* = −2.33, *SE* = 0.34, Wald (1) = 45.82, *p* < .001, OR = 0.10. Step 1 significantly improved the goodness of fit compared to a model containing only the constant, *χ^2^* (1) = 54.85, *p* < .001. Gaming experience, performance, and motivational variables were not significantly associated with dismissal during Task #3. Step 2 did not significantly improve the goodness of fit, *χ^2^* (6) = 2.32, *p* = .889. Consistent with expectations, demand‐related action orientation was associated with dismissal during a task, *B* = 0.10, *SE* = 0.05, Wald (1) = 4.44, *p* = .035, OR = 1.11. One unit increase in demand‐related action orientation corresponded to a 1.11 times (11%) higher probability of dismissal during a task. Step 3 significantly improved the goodness of fit, *χ^2^* (1) = 4.58, *p* = .032. Overall, the model explained 35% of variance in dismissal versus nondismissal. Entered threat‐ instead of demand‐related action orientation in Step 3 did not yield a significant effect, *B* = 0.04, *SE* = 0.05, Wald (1) = 0.71, *p* = .400, OR = 1.04. Thus, the effect during Task #3 was specific for demand‐related action orientation and occurred when controlling for dismissal after Task #1, gaming experience, performance, and task motivation.

**Figure 3:**
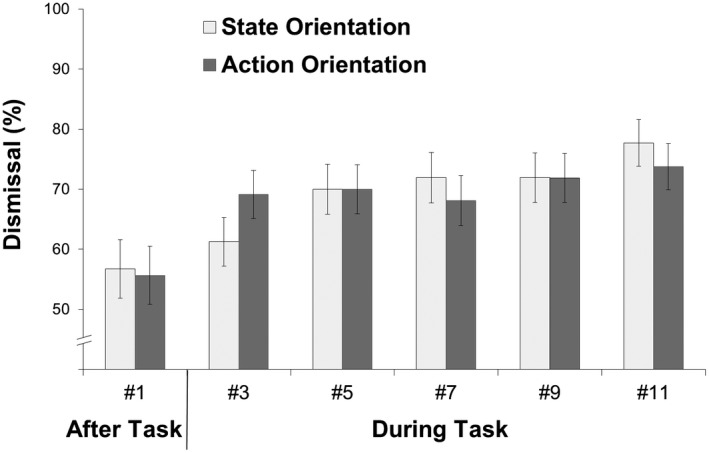
Initiative (dismissal of notification) as a function of demand (interruption during vs. after task) and demand‐related action–state orientation in Study 3 (*N* = 210; notification timeout: 10 s)

In binary logistic regression analyses with dismissal versus nondismissal during Tasks #5–#11 as a dependent variable, there were no significant effects in Steps 2 and 3. Specifically, participants with higher action orientation were not more likely to dismiss the notifications during Task #5, *B* = 0.03, *SE* = 0.05, Wald (1) = 0.31, *p* = .575, OR = 1.03, Task #7, *B* = 0.02, *SE* = 0.05, Wald (1) = 0.18, *p* = .676, OR = 1.02, Task #9, *B* = 0.03, *SE* = 0.05, Wald (1) = 0.54, *p* = .464, OR = 1.03, and Task #11, *B* = 0.01, *SE* = 0.05, Wald (1) = 0.07, *p* = .781, OR = 1.01. Thus, repetition of task interruptions led to fast adaptation. To further illustrate the finding (see Figure 3), we graphed dismissal rates during and after tasks using a median split to classify participants as state‐oriented (*n* = 104, scores 0–7) and action‐oriented (*n* = 106, scores 8–12).

#### 4.2.4 Latency

When setting latencies for nondismissal to 10 s, average dismissal latencies were *M* = 4.84 s (*SD* = 3.08). As listed in Table 3, the regression analysis for dismissal latencies during Task #3 showed a significant baseline effect, *β* = 0.48, *t*(1, 208) = 7.87, *p* < .001. There were no significant effects of gaming experience, performance, and motivational variables. Consistent with expectations, demand‐related action orientation was associated with faster dismissal of the notification during Task #3, *β* = −0.13, *t*(1, 201) = −2.01, *p* = .046, and accounted for additional variance, ∆*R* ^2^ = 0.015, ∆*F*(1, 201) = 4.03, *p* = .046. There was no significant effect when entering threat‐ instead of demand‐related action orientation, *β* = −0.06, *t*(1, 201) = −0.85, *p* = .397. In regression analyses with dismissal latencies during Tasks #5–#11 as a dependent variable, there were no significant effects in Steps 2 and 3. Findings indicate that this more continuous measure of initiative yielded similar effects to dismissal versus nondismissal.

When including only the 88 participants who dismissed all notifications, average dismissal latencies were *M* = 2.29 s (*SD* = 0.70). As listed in Table 3, in this subsample, the same regression analysis for dismissal latencies during Task #3 showed a significant baseline effect, *β* = 0.25, *t*(1, 86) = 2.38, *p* = .019. There were no further significant effects. Specifically, demand‐related action orientation was not associated with *slower* (and descriptively even faster) dismissal during Task #3, *β* = −0.22, *t*(1, 79) = −1.95, *p* = .055. Regression analyses with dismissal latencies during Tasks #5–#11 as a dependent variable did not show any significant effects in Steps 2 and 3. Findings do not suggest that action‐oriented participants engaged in more deliberate decision processes.

### 4.3 Discussion

In Study 3, we again replicated our central finding. Action orientation was associated with higher probability to dismiss the notification during a task (under demand), when controlling for dismissal after a task (no demand). In addition, after the first repetition, state‐oriented participants had effectively adapted to the demand and were as likely to dismiss notifications during tasks as were action‐oriented participants.

## 5 GENERAL DISCUSSION

Daily hassles are the annoying, frustrating, or stressful demands that occur through our daily interactions with our environment (Kanner, Coyne, Schaefer, & Lazarus, 1981). Daily hassles include unexpected events such as dealing with a sick child, a traffic jam on the way to work, or a phone call during an important meeting. In the present studies, we investigated the impact of an even smaller demand on individuals' ability to initiate goal‐directed action: an interrupting system notification while playing a fun computer game. Participants simply had to click a button to continue playing the game. In three studies, we found that action orientation predicted higher initiative (i.e., higher probability of dismissal and lower dismissal latencies) when interrupted *during* game rounds (under demand), controlling for initiative when interrupted *after* game rounds (no demand). Our findings extend classical findings of Ovsiankina (1928) that individuals tend to resume interrupted activities. In the present studies, we show that the tendency to continue with an interrupted goal‐related activity is stronger for action‐ than for state‐oriented individuals under demand. In other words, the classical Ovsiankina effect is moderated by action–state orientation.

Note that action‐oriented participants' initiative under demand was observed across three studies, across two different time frames for initiative (60 s in Study 1; 10 s in Studies 2 and 3), and across two different measures for initiative (dismissal vs. nondismissal; dismissal latencies). This methodological convergence increases confidence in the robustness of our findings. Furthermore, the findings remained significant when controlling for task ability (gaming experience and performance) and several indicators of task motivation. Thus, state‐oriented participants' lower probability of dismissal under demand cannot be easily explained by less familiarity with computers, lower interest in the game, or greater tension that may call for taking a break. Dismissal latencies of the subsamples of participants who dismissed notifications do not indicate that state‐ and action‐oriented participants differed in deliberate decision processes that may have biased them toward dismissal or nondismissal. Instead, the findings are consistent with the large body of research indicating that state‐ and action‐oriented individuals differ in the *self‐regulatory ability* to initiate goal‐directed action under demanding conditions (Kazén et al., 2008; Koole et al., 2012; Kuhl, 1981, 2000, 2001; Kuhl & Beckmann, 1994b).

In the context of our studies, waiting a few seconds longer to continue a fun game may not appear dramatic or disadvantageous. However, our differential effect unravels a more general mechanism: A negligible demand suffices to hamper initiative for participants who have lower action orientation (i.e., state orientation). This may have important consequences in other contexts and widen the intention–behavior gap. Therefore, it is important to elaborate why exactly interruptions during game rounds are so demanding. One possible (affective) explanation is that they elicit more feelings of annoyance and frustration (as Bailey & Konstan, 2006, show), which interfere with the initiation of goal‐directed action. Previous findings show that state‐oriented individuals are less able to overcome feelings of frustration (Baumann et al., 2007; Brunstein, 2001; Koole & Jostmann, 2004). Another possible (cognitive) explanation is that individuals form an intention to complete the interrupted task, which creates cognitive load. The classical work by Zeigarnik (1927) shows that intentions for uncompleted tasks have a privileged status in working memory. In an advanced paradigm, Goschke and Kuhl (1993) found that state‐oriented participants exhibit an overly strong intention superiority effect.

The theory of Personality Systems Interactions (PSI; Kuhl, 2000, 2001) allows the integration of the affective and cognitive explanations, and elaborates why the activation of an intention produces a paradoxical deficit in the initiation of corresponding action. According to PSI theory, intentions are formed and maintained in a specialized cognitive system (i.e., *intention memory*) whenever an action cannot be carried out immediately through automatic behavioral programs (Goschke & Kuhl, 1993). Activation of intention memory reduces positive affect (and vice versa) and inhibits the behavioral output system linked to it. This decoupling of intentions from action is adaptive for analytical problem solving, planning, and sequencing of action steps. However, it can also result in hesitation, rumination about unfulfilled goals, and an intention–behavior gap (Ruigendijk et al., 2018). Intention memory is recoupled with its behavioral output system when positive affect (either self‐generated or externally provided) indicates that an opportunity for successful action is encountered (Kazén et al., 2008; Kuhl & Kazén, 1999).

Thus, PSI theory suggests that state‐oriented individuals' hesitation to dismiss a notification that interrupts a game round is due to their tendency to activate intentions (e.g., the intention to complete the game round) and their lower ability to self‐generate the positive affect needed to actually carry them out. Both mechanisms are mutually intensifying and have been shown to be a risk factor in the development, chronicity, and recurrence of depression (Kuehner & Huffziger, 2013; Kuhl & Beckmann, 1994b; Kuhl & Helle, 1986). Most importantly, these mechanisms indicate a volitional rather than motivational deficit: State‐oriented individuals are not less motivated to put intentions into action but they are less able to initiate action. This has important implications for the practice. Whereas motivation can be modified by enhancing incentives and subjective control beliefs (e.g., Atkinson, 1957; Bandura, 1997), this does not suffice when dealing with self‐regulatory deficits (see also Wolf, Herrmann, & Brandstätter, 2018). Instead, it takes interventions that either compensate self‐regulatory deficits (e.g., external control) or train and develop self‐regulatory abilities.

The present findings show that repetition can mitigate the effects of demands. When interrupted a second time during a game round, state‐oriented participants already adapted to the demand. This easy solution may suffice for the present small‐scale demand. However, repetition does not promote self‐regulatory abilities that state‐oriented individuals need to develop in the long run. Self‐regulation may be trained, for example, through “mental contrasting” (Oettingen, Pak, & Schnetter, 2001)—a technique that practices oscillating between reduced and restored positive affect (e.g., positive fantasies about the future and present difficulties). Furthermore, self‐regulation develops in responsive relationships (Kuhl, 2000; Kuhl & Keller, 2008). If parents, teachers, or colleagues respond promptly and adequately to self‐expressions (e.g., encouragement in case of frustration) the emotion‐regulatory effect becomes conditioned to the self. Thereby, the originally externally supported emotion regulation gradually turns into self‐regulation (e.g., the ability to self‐generate positive affect).

## 6 LIMITATIONS AND FUTURE PERSPECTIVES

Of course, the present research leaves many questions open for future research. First, although the effect sizes for action–state orientation were within the typical range of personality correlates, they were rather small given that the present paradigm is at the heart of the construct. It is possible that our behavioral assessment had too much noise and that interruptions were too subtle. Future studies can make interruptions during a game round more salient by better highlighting the start and end of game rounds. However, even the minor differences in initiative we observed can have major impact if we consider their compound effect over time. Our manipulation was subtle—simply dismissing a notification—but is persistent across digital interactions on computers, smartphones, and gaming consoles. A small lack of initiative exhibited throughout frequent digital interactions can add up to a larger impact.

Second, we assessed participants' motivation for the game only at the end of the experiment. Future studies could assess participants' motivation to dismiss the notification and continue with the game directly after being interrupted to further examine a motivational explanation of our findings. Furthermore, the overall high probability of dismissal (70%–92%) suggests that our setting has induced an “implemental mind set” that is well suited to tease out volitional determinants of initiative (Harmon‐Jones & Harmon‐Jones, 2002). Settings in which participants are more uncertain whether they want to continue playing, in contrast, may induce a “decisional mind set” that is better suited to tease out motivational determinants of initiative (Heckhausen & Gollwitzer, 1987). Thus, it would be informative to generalize our findings across different tasks and different settings (e.g., mind sets).

In a similar vein, we did not directly measure the presumed mechanisms behind state‐oriented individuals' initiation deficit: higher activation of intentions and lower positive affect. However, these functional features of demand‐related state orientation are well documented in the literature (Brunstein, 2001; Goschke & Kuhl, 1993; Kuhl, & Beckmann, 1994b). Furthermore, we experimentally manipulated the load on intention memory: uncompleted tasks have a special status in memory (Goschke & Kuhl, 1993). In future studies, it would nevertheless be informative to have a sensitive online measure of positive affect.

In the present studies, we reduced the demand level to a minimum and tested initiative on a micro‐level of analysis. This methodological strength may entail an ecological limitation: One may question whether enduring a 10 s wait after an interruption during a video game has any implications for psychological functioning in general. Although a minor hassle, their frequency of occurrence—40% of days include a daily stressor (Almeida, 2005)—make them important to consider. In fact, evidence suggests that frequent minor demands, such as sitting in traffic, may have a similar impact on health as infrequent major demands, such as a divorce (DeLongis, Coyne, Dakof, Folkman, & Lazarus, 1982). Furthermore, previous research reveals the same differential effects for action–state orientation across small‐ and large‐scale demands. Thus, the present findings have implications for broader psychological functioning.

Finally, we found that repetition mitigated the impact of demand on state‐oriented individuals' initiative (Study 3). However, participants have little control over the frequency of interruptions in daily life. Future studies should look for mitigating factors that are less externally controlled and more easily applied as a self‐help technique. For example, visualising an accepting (vs. demanding) person and thinking of similarities (vs. differences) with a close other has been found to reduce the detrimental effects of stress on state‐oriented individuals' well‐being (Chatterjee, Baumann, & Koole, 2017; Koole & Jostmann, 2004). It would be informative to test whether focusing on relatedness may also foster initiative and whether state‐oriented individuals are able to activate a focus on relatedness by themselves.

## 7 CONCLUDING REMARKS

Task interruptions occur frequently in daily life (Almeida, 2005), but when experienced frequently, persistently, and repeatedly, even minor interruptions can have major impact. Although only a minor hassle, they are sufficiently demanding to reduce initiative and widen the intention–behavior gap in state‐ but not in action‐oriented individuals. This differential effect unravels an important mechanism. A large body of work has suggested that action–state orientation is about the self‐regulatory ability to initiate under demand (Diefendorff et al., 2006; Kuhl, 2000, 2001; Kuhl & Beckmann, 1994b). In the present studies, we rule out the potential mediating effects of ability and motivation for the primary task, the need for a break due to in‐game tension, and initiative under no demand. Thus, we can conclude that our state‐oriented participants are not less motivated to resume interrupted tasks but less able to self‐motivate and initiate goal‐directed action.

The differential ability to initiate under minor demands has major implications. State‐oriented individuals tend to activate intentions under demand, but are less capable of generating the positive affect needed to carry them out (Goschke & Kuhl, 1993). This tendency has been shown to be a risk factor for depression (Kuehner & Huffziger, 2013), suggesting implications for the well‐being of state‐oriented individuals who are less able to initiate under the frequent minor demands of daily life. Although we show how repeated exposure to a single demand can prompt initiative, demands from daily hassles are not predictable, and external control through prompting does not promote the self‐regulatory abilities that state‐oriented individuals need to develop in the long run. Future work can build on our findings to evaluate training programs that specifically target self‐regulatory abilities.

## CONFLICTS OF INTEREST

The authors declared no potential conflicts of interest with respect to the research, authorship, and/or publication of this article.
